# Convergence of soil microbial properties after plant colonization of an experimental plant diversity gradient

**DOI:** 10.1186/s12898-016-0073-0

**Published:** 2016-04-07

**Authors:** Katja Steinauer, Britta Jensen, Tanja Strecker, Enrica de Luca, Stefan Scheu, Nico Eisenhauer

**Affiliations:** German Centre for Integrative Biodiversity Research (iDiv) Halle-Jena-Leipzig, Deutscher Platz 5e, 04103 Leipzig, Germany; Institute of Biology, Leipzig University, Johannisallee 21, 04103 Leipzig, Germany; J. F. Blumenbach Institute of Zoology and Anthropology, Georg-August-University Göttingen, Berliner Straße 28, 37073 Göttingen, Germany; Institute of Evolutionary Biology and Environmental Studies, University of Zurich, Zurich, 8057 Switzerland

**Keywords:** Jena Experiment, Plant colonization, Microbial biomass, Plant diversity, Plant coverage

## Abstract

**Background:**

Several studies have examined the effects of plant colonization on aboveground communities and processes. However, the effects of plant colonization on soil microbial communities are less known. We addressed this gap by studying effects of plant colonization within an experimental plant diversity gradient in subplots that had not been weeded for 2 and 5 years. This study was part of a long-term grassland biodiversity experiment (Jena Experiment) with a gradient in plant species richness (1, 2, 4, 8, 16, and 60 sown species per plot). We measured plant species richness and productivity (aboveground cover and biomass) as well as soil microbial basal respiration and biomass in non-weeded subplots and compared the results with those of weeded subplots of the same plots.

**Results:**

After 2 and 5 years of plant colonization, the number of colonizing plant species decreased with increasing plant diversity, i.e., low-diversity plant communities were most vulnerable to colonization. Plant colonization offset the significant relationship between sown plant diversity and plant biomass production. In line with plant community responses, soil basal respiration and microbial biomass increased with increasing sown plant diversity in weeded subplots, but soil microbial properties converged in non-weeded subplots and were not significantly affected by the initial plant species richness gradient.

**Conclusion:**

Colonizing plant species change the quantity and quality of inputs to the soil, thereby altering soil microbial properties. Thus, plant community convergence is likely to be rapidly followed by the convergence of microbial properties in the soil.

**Electronic supplementary material:**

The online version of this article (doi:10.1186/s12898-016-0073-0) contains supplementary material, which is available to authorized users.

## Background

Human-induced global change is leading to worldwide changes in plant community assembly resulting in profound impacts on ecosystem functions [1, 2]. Gaining more knowledge about the mechanisms that influence biodiversity, compositional stability of plant communities, and resistance against plant colonization provide essential information to evaluate the consequences of biodiversity loss and the subsequent changes in ecosystem functions.

Generally, plant diversity increases the stability of community biomass in biodiversity experiments [3–5]. Presumably, this is due to more complete exploitation of resources with increasing plant diversity [6]. Therefore, more diverse plant communities are more resistant to the colonization of species than less diverse plant communities [7–9] and/or are more likely to contain better competitors for available resources [10]. Newly colonizing plant species must survive and grow on resources not consumed by resident plant species [7]. The prerequisite for successful plant colonization thus might be complementary resource requirements compared to the resident plant species [11]. Therefore, colonization success of a plant species is higher when its functional traits are most different from the functional traits of the resident species [12–14]. Consistent with this expectation, higher species richness and functional complementarity were shown to increase plant biomass production in experimental studies [15, 16] due to a more complete use of available resources [17] leaving fewer vacant niches for colonizers [7, 13]. The composition and functioning of plant communities are closely linked to belowground communities and processes [18]. Colonizing plant species entering a resident community affect the biogeochemistry of ecosystems [19], alter the rate of nutrient cycling [20], and induce a shift in the structure of rhizosphere microbial communities [21], e.g., by accumulating specific pathogens in their rhizosphere [22].

Previous studies showed that the biomass and the activity of soil microorganisms increase significantly with increasing plant diversity [23–25]. The underlying mechanisms are enhanced net primary productivity, soil carbon inputs via rhizodeposition, and decomposition of plant biomass at high plant diversity [26, 27]. Different plant species, including colonizer species, release different organic compounds that change the rhizosphere conditions affecting the microbial community structure, abundance, and activity [28]. Some newly colonizing plant species might produce chemical compounds that are novel to the resident plant species, thereby having unique effects on soil microbial properties. Therefore, shifts in plant community composition and diversity due to the colonization of plant species may affect soil microbial community composition, biomass, and functions [21].

**Fig. 1:**
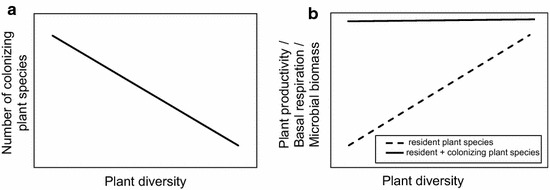
Conceptual figures showing the three hypothesis. **a** The number of colonizing plant species was expected to decrease with increasing plant diversity of resident species. **b** Plant biomass production, soil basal respiration, and microbial biomass increases were expected to increase with increasing plant diversity (*dashed line*) in weeded subplots, while those variables will become similar across all initial experimental plant diversity levels after plant colonization in non-weeded subplots (*solid line*). The reader should note that the conceptual figures are graphically and qualitatively depicting the hypotheses. For simplicity, we depicted linear relationships between the variables. However, these *lines* are not based on empirical data, and we did not necessarily expect linear relationships between variables

Given that there is still a need to advance knowledge about the consequences of plant colonization for soil microbial communities, we studied the effect of plant colonization of an experimental plant diversity gradient. In this split-plot experiment, one set of subplots were not weeded, while the other set of subplots were weeded. The present study was part of an established long-term grassland biodiversity experiment (Jena Experiment) with a gradient in plant diversity (1, 2, 4, 8, 16, and 60). We hypothesized that species-rich plant communities are more resistant to colonizing plant species than species-poor plant communities (hypothesis 1; Fig. 1a) [29]. Due to the colonization of functionally dissimilar plant species, we expected that plant diversity and productivity will become similar across all initial experimental plant diversity levels after plant colonization [7, 30]. Further, we expected soil respiration and microbial biomass to increase with higher plant diversity in weeded subplots (hypothesis 2, Fig. 1b, solid line) [25, 31]. As a result of plant colonization in non-weeded subplots, we expected the effects of the initial plant diversity gradient to disappear due to a homogenization of the quality and quantity of plant material entering the soil [32], thereby inducing a convergence of soil microbial properties (hypothesis 3, Fig. 1b, dotted line).

## Methods

The study was conducted as part of the Jena Experiment, a long-term grassland biodiversity experiment in Jena, Germany [33]. A plant diversity gradient (1, 2, 4, 8, 16, and 60 species) was established in 2002 on 82 plots (two monoculture plots had to be given up over the course of the experiment due to very low coverage of the target species, which resulted in 80 plots for the present study). The species pool of the experiment consists of 60 plant species categorized into four functional groups (grasses, legumes, tall and small herbs; see Additional file 1: Table S1 for complete species list of plant species pool). Monocultures, two-, four-, and eight-species mixtures were replicated 16 times, 16-species-mixtures were replicated 14 times and the complete species pool of 60 species was replicated four times. Sowing density amounted to 1000 viable seeds per m^2^ divided equally among species. Plant community composition was maintained by weeding all experimental plots three times per year (May, July, and September) to remove all non-target species. Here, we use the terms “resident” for plant species initially sown in experimental plots (i.e., target plant species) and “colonizing” for plant species not sown originally in the plots (i.e., non-target plant species).

We established two independent experiments (A and B) differing in length of plant colonization (experiment A: 2 years, experiment B: 5 years) within 80 main plots resulting in 160 subplots for each experiment in a split-plot design. Therefore, the sown plant species combination and planting density was the same in both experiments. In experiment A, two subplots of 1 × 1 m were established in autumn 2009. In one subplot, regular weeding was continued during the entire study, whereas in the other subplot, weeding was stopped for approximately 2 years allowing for plant colonization. Using a metal corer, five soil samples (diameter 2 cm, 10 cm deep) per subplot were randomly taken in June 2011. Additionally, plant cover (%) of resident and colonizing plant species were estimated using a modified decimal Londo scale [34] and used as a proxy for plant productivity. Numerical values for species cover were coded as 1 (<1 %), 2 (1–5 %), 10 (6–15 %), 20 (16–25 %), 30 (26–35 %), 40 (36–45 %), 50 (46–55 %), 60 (56–65 %), 70 (66–75 %), 80 (76–85 %), and 90 (>85 %). Subplots of experiment A subplots were given up after this sampling campaign and therefore they were no more available for further measurements.

In experiment B, new subplots (5 × 3 m) were established in 2009, following the same format as in experiment A: in one subplot, regular weeding was continued, and in the other subplot, weeding was stopped. Like experiment A, five soil samples per subplot were randomly taken within 1 × 1 m in May 2014, i.e., after 5 years of plant colonization in the non-weeded subplots. Here, plant productivity was measured in two 0.1 m^2^ plots as aboveground plant biomass (g m^−2^).

In both experiments, soil samples were pooled, homogenized, sieved (2 mm), and approximately 5 g (fresh weight) of each soil sample was used for the measurement of soil microbial biomass and respiration. Microbial respiration (µL O_2_ h^−1^ g^−1^ soil dry mass) was measured as mean of the O_2_ consumption rates of 14–24 h after the start of the measurements using an O_2_-microcompensation apparatus [35]. Soil microbial biomass C (µg C g^−1^ soil dry mass) was measured by substrate-induced respiration (SIR) after the addition of d-glucose [36]. Due to soil sieving fungal hyphae are broken up and therefore, this method mainly measures the respiration and biomass of soil bacteria. Gravimetric soil water content (%) was determined as the difference in percentages of fresh vs. dry soil (dried at 70 °C for 24 h).

First, we used General Linear Models (GLM) for a split-plot design (t-values with Satterthwaite approximation) to test effects of plant diversity (PD; manipulated at the plot level), plant colonization (COL; weeded vs. non-weeded subplots), and the interaction of plant diversity and plant colonization (PD × COL) on microbial respiration, microbial biomass (both experiments), plant cover (experiment A), plant biomass (experiment B), and number of colonizing plant species (both experiments). Second, we used a linear mixed effect model (t-values with Satterthwaite approximation) to test the effects of sown plant diversity (PD) in weeded and non-weeded plots independently on microbial respiration, microbial biomass (both experiments), plant cover (experiment A only), and plant biomass (experiment B only). Both analyses were performed using the core functions within the R statistical environment (R Development Core Team 2013) and the lme4 package [37].

## Results

**Fig. 2:**
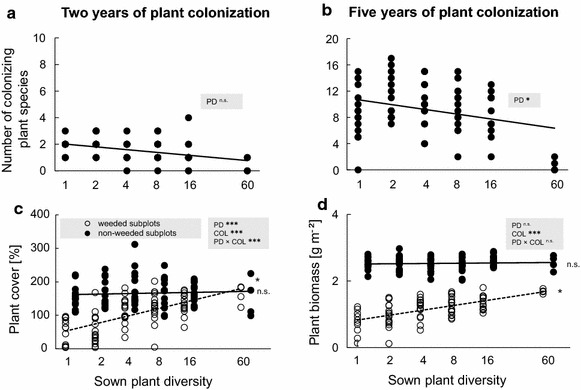
Plant colonization effects on plant cover and plant biomass. Number of colonizing plant species after **a** 2 years and **b** 5 years. **c** Plant cover [%] after 2 years and **d** plant biomass [g m^−2^] after 5 years. In **c** and **d** *dashed lines* and *open circles* display plant cover and biomass with resident plant species of weeded subplots, respectively, and *solid lines* and *circles* *display* plant cover and biomass with resident plant species plus colonizing plant species of non-weeded subplots, respectively. *** P < 0.001, ** P < 0.01, * P < 0.05, (*) P < 0.1, ns P > 0.1

In both experiments, the number of newly colonizing plant species decreased with increasing plant diversity (Table 1; Fig. 2a, b; Additional file 2: Figure S2a, b; for complete species list of colonizing plant species see Additional file 1: Table S1). Two years after plant colonization (experiment A), on average 2 plant species colonized the monocultures compared to 10 plant species after 5 years of plant colonization (experiment B). Overall, in experiment B the number of colonizing plant species increased ~ fourfold per plant diversity level in comparison to the results of experiment A (after 2 years).

**Table 1:** LM table of t- and P-values on the effects of plant diversity (PD: 1, 2, 4, 8, 16, and 60 plant species) in weeded or non-weeded plots on number of colonizing plant species, plant cover (2011), plant biomass (2014), soil basal respiration, soil microbial biomass and soil water content of 2011 and 2014

	Number of colonizing plant species			Plant cover			Soil basal respiration			Soil microbial biomass			Soil water content		
	df	t value	P value	df	t value	P value	df	t value	P value	df	t value	P value	df	t value	P value
2011															
Weeded				1, 77.01	**4.13**	**<0.001**	1, 72.18	**4.29**	**<0.001**	1, 74.35	**3.57**	**<0.001**	1, 74.08	**4.58**	**<0.001**
Non-weeded	1, 77.00	−*1.93*	*0.057*	1, 76.99	−0.30	0.767	1, 71.18	0.80	0.428	1, 72.33	1.14	0.256	1, 73.10	3.07	**0.003**

	Number of colonizing plant species			Plant biomass			Soil basal respiration			Soil microbial biomass			Soil water content		
	df	t value	P value	df	t value	P value	df	t value	P value	df	t value	P value	df	t value	P value
2014															
Weeded				1, 74.34	**5.57**	**<0.001**	1, 74.09	**4.26**	**<0.001**	1, 74.01	**2.74**	**0.008**	1, 74.10	5.30	**<0.001**
Non-weeded	1, 74.05	**−2.14**	**0.036**	1, 76.04	1.09	0.281	1, 71	0.23	0.815	1, 68.48	0.08	0.938	1, 70.09	1.29	0.202

Significant results (P < 0.05) are highlighted in bold and marginally significant results (P < 0.10) are given in italics

### Experiment A—effects of 2 years of plant colonization

**Fig. 3:**
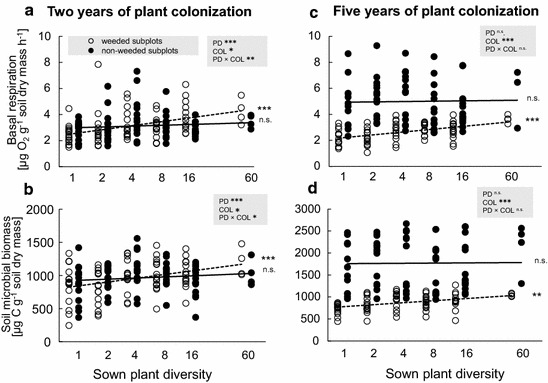
Plant colonization effects on soil microbial properties. Basal respiration [µg O_2_ g^−1^ soil dry mass h^−1^] **a** 2 and **c** 5 years, and soil microbial biomass [µg C g^−1^ soil dry mass] **b** 2 and **d** 5 years after colonization by plant species. *Dashed lines* and *open circles* display basal respiration and soil microbial biomass with resident plant species of weeded subplots, respectively, and *solid lines* and *circles display* basal respiration and soil microbial biomass with resident plant species plus colonizing plant species of non-weeded subplots, respectively. ***P < 0.001, **P < 0.01, *P < 0.05, (*) P < 0.1, ns P > 0.1

In experiment A, plant cover in weeded subplots was significantly higher in species-rich plant communities of resident plant species than in species-poor ones (Table 1; Fig. 2c; Additional file 2: Figure S2c). After 2 years of plant colonization, total plant cover was similar across all sown plant diversity levels in non-weeded subplots (Table 1), resulting in a significant interaction of plant diversity and plant colonization (Table 2). In weeded subplots, increasing plant diversity significantly increased both soil basal respiration (Table 1; Fig. 3a; Additional file 3: Figure S3a) and microbial biomass (Table 1; Fig. 3b; Additional file 3: Figure S3b). In non-weeded plots, basal respiration and soil microbial biomass slightly increased in species-poor plant communities and slightly declined in species-rich plant communities after plant colonization in comparison to weeded plots, rendering the plant diversity effect on soil microbial properties insignificant (Tables 1, 2). Gravimetric soil water content increased significantly with increasing plant diversity in both weeded and non-weeded subplots (Tables 1, 2).

**Table 2:** GLM table of t- and P-values on the effects of plant diversity (PD: 1, 2, 4, 8, 16, and 60 plant species) and plant colonization (COL) on plant cover (2011), plant biomass (2014), soil basal respiration, soil microbial biomass and soil water content of 2011 and 2014

	Plant cover			Soil basal respiration			Soil microbial biomass			Soil water content		
	df	t value	P value	df	t value	P value	df	t value	P value	df	t value	P value
2011												
PD	1, 145.61	**4.46**	**<0.001**	1, 140.86	**4.15**	**<0.001**	1, 132	**3.67**	**<0.001**	1, 145.30	**4.79**	**<0.001**
COL	1, 77	**9.42**	**<0.001**	1, 74.25	**2.06**	**0.043**	1, 76.33	**2.06**	**0.043**	1, 75.18	0.37	0.714
PD × COL	1, 77	**−3.84**	**<0.001**	1, 74.69	**−2.64**	**0.010**	1, 76.42	**−2.25**	**0.028**	1, 75.29	−1.49	0.139

	Plant Biomass			Soil basal respiration			Soil microbial biomass			Soil water content		
	df	t value	P value	df	t value	P value	df	t value	P value	df	t value	P value
2014												
PD	1, 152.79	0.61	0.543	1, 147.77	*1.90*	*0.060*	1, 143.35	1.11	0.270	1, 137.59	**5.85**	**<0.001**
COL	1, 77.94	**11.45**	**<0.001**	1, 78.53	**7.50**	**<0.001**	1, 77.23	**8.78**	**<0.001**	1, 73.92	**−6.37**	**<0.001**
PD × COL	1, 77.45	0.67	0.505	1, 77.27	−1.12	0.268	1, 76.00	−0.72	0.474	1, 73.72	**−3.79**	**<0.001**

Significant results (P < 0.05) are highlighted in bold and marginally significant results (P < 0.10) are given in italics

### Experiment B—effects of 5 years of plant colonization

In experiment B, plant biomass increased with increasing plant diversity in weeded subplots, however this positive relationship disappeared in non-weeded subplots (Table 1; Fig. 2d; Additional file 2: Figure S2d). Despite these different trends, the interaction effect of plant diversity and plant colonization was not significant (Fig. 2d; Additional file 2: Figure S2d). Generally, plant biomass was significantly higher in non-weeded subplots than in weeded subplots (+120 %). In weeded subplots, soil basal respiration (Table 1; Fig. 3c; Additional file 3: Figure S3c) and microbial biomass (Table 1; Fig. 3d; Additional file 3: Figure S3d) increased significantly with increasing sown plant diversity. However, basal respiration and soil microbial biomass were not affected by sown plant diversity in non-weeded subplots, although the interaction effect of plant diversity and plant colonization was not significant (Tables 1, 2; Fig. 3c, d; Additional file 3: Figure S3c, d). Both basal respiration (+90 %) and soil microbial biomass (+104 %) were significantly higher in non-weeded subplots than in weeded subplots. Soil water content increased significantly with increasing plant diversity in weeded subplots but was not significantly affected by plant diversity after plant colonization in non-weeded subplots (Tables 1, 2).

## Discussion

Total productivity (plant coverage and biomass, respectively) of resident plant communities increased with increasing plant diversity in both experiments, confirming hypothesis 1 [30]. In addition, numbers of colonizing plant species typically were high in species-poor plant communities and decreased with increasing plant diversity. In line with our findings, previous studies suggested that diverse plant communities better resist plant colonization than less diverse communities [29, 38] due to lower levels of available resources [39]. Consequently, less resources are available for potential new colonizer species [40, 41]. Furthermore, there is evidence that large niche overlap between resident and colonizer species increases resistance against colonization [42, 43]. Generally, empty niche space is assumed to decline with increasing species richness [7, 13]. Thus, a diverse plant community should be more resistant to colonizer plant species when depending on similar resources [40, 44].

Importantly, colonizing plant species may change the quantity and quality of inputs to soil [45, 46], which has the potential to alter soil microbial functions and processes. In line with hypothesis 2, soil basal respiration and microbial biomass increased with increasing plant diversity in weeded subplots of both experiments [31]. Type and number of plant species present have considerable influence on the functions and diversity of soil microorganisms [27, 47]. Soil microorganisms are involved in processes like decomposition and nutrient mineralization and their community composition and abundances have been shown to vary with plant species [48]. Since increasing plant biomass production and release of rhizodeposits is associated with higher availability and diversity of plant-derived resources, we suggest that plant colonization positively influenced soil microbial properties in both experiments [26, 46]. Moreover, root morphological characteristics, litter types, and plant tissue qualities may affect the biomass of soil microorganisms [49]. Therefore, an increase of plant species richness, presumably resulting in a convergence of plant community composition [38] in non-weeded subplots in both experiments, may have induced a shift in soil microbial properties equalizing the effects of the initially sown plant diversity gradient, confirming hypothesis 3. After 2 and 5 years of plant colonization, basal respiration and soil microbial biomass increased in species-poor plant communities in non-weeded subplots in comparison to weeded subplots. However, in species-rich plant communities soil microbial respiration and biomass decreased after 2 years of plant colonization, which is hard to explain. In contrast, after 5 years of plant colonization, basal respiration and soil microbial biomass were considerably higher across all plant diversity levels in non-weeded subplots, but particularly at low plant diversity. This indicates that time could play a crucial role in the establishment of plant diversity effects on soil microbial properties. Previous studies [31, 50] showed that several years are required to display significant plant diversity effects on soil microbial biomass due to the slow accumulation of plant-derived resources in the soil over time [51, 52]. Although such a temporal effect could explain the differences between the two experiments in the present study, please note that we only have two sampling dates, which does not allow us to infer temporal trends.

## Conclusion

Our study highlights the consequences of plant colonization for resident plant communities and soil microbial properties. The results confirmed previous findings that experimental communities with higher numbers of resident plant species are more resistant to colonization than species-poor ones [30, 38]. Further, the present results show that plant community convergence induces the convergence of microbial properties in the soil. Colonizing plant species are likely to change the quantity and quality of inputs to the soil, thereby altering soil microbial functions and processes. Future studies should investigate the potential convergence of soil microbial community composition and multiple microbial functions. Further, it remains to explore specific plant traits effects on particular microbial taxa and functions in the soil, and if novel plant traits in a colonized plant community and convergence of the functional composition of plant communities are the underlying mechanisms of the observed convergence of soil microbial properties.

## Additional files

10.1186/s12898-016-0073-0 Plant species of weeded subplots (resident plant species) and non-weeded subplots (colonizing plant species). Colonizing plant species are divided into internal (belonging to the species pool of the resident plant species) and external (not belonging to the species pool of the resident plant species) plant species.
10.1186/s12898-016-0073-0 Plant colonization effects on plant cover and plant biomass. Mean values with confidence intervals of colonizing plant species after (a) two years and (b) five years. (c) Mean values with confidence intervals of plant cover [%] after two years and (d) plant biomass [g m^−2^] after five years. In c) and d) circles display plant cover and biomass of resident plant species of weeded subplots, respectively, and open circles display plant cover and biomass with resident plant species plus colonizing plant species of non-weeded subplots, respectively.
10.1186/s12898-016-0073-0 Plant colonization effects on soil microbial properties. Mean values with confidence intervals of basal respiration [µg O_2_ g^−1^ soil dry mass h^−1^] (a) two and (c) five years, and soil microbial biomass [µg C g^−1^ soil dry mass] (b) two and (d) five years after colonization by plant species. Circles display basal respiration and soil microbial biomass with resident plant species of weeded subplots, respectively, and open circles display basal respiration and soil microbial biomass with resident plant species plus colonizing plant species of non-weeded subplots, respectively.
