# Supplementary figures and images for: Convergence of soil microbial properties after plant colonization of an experimental plant diversity gradient
Source: BMC Ecol. 2016 Apr 7;16:19. doi: 10.1186/s12898-016-0073-0 (PMC4825091; doi:10.1186/s12898-016-0073-0)

# Two years of plant colonization

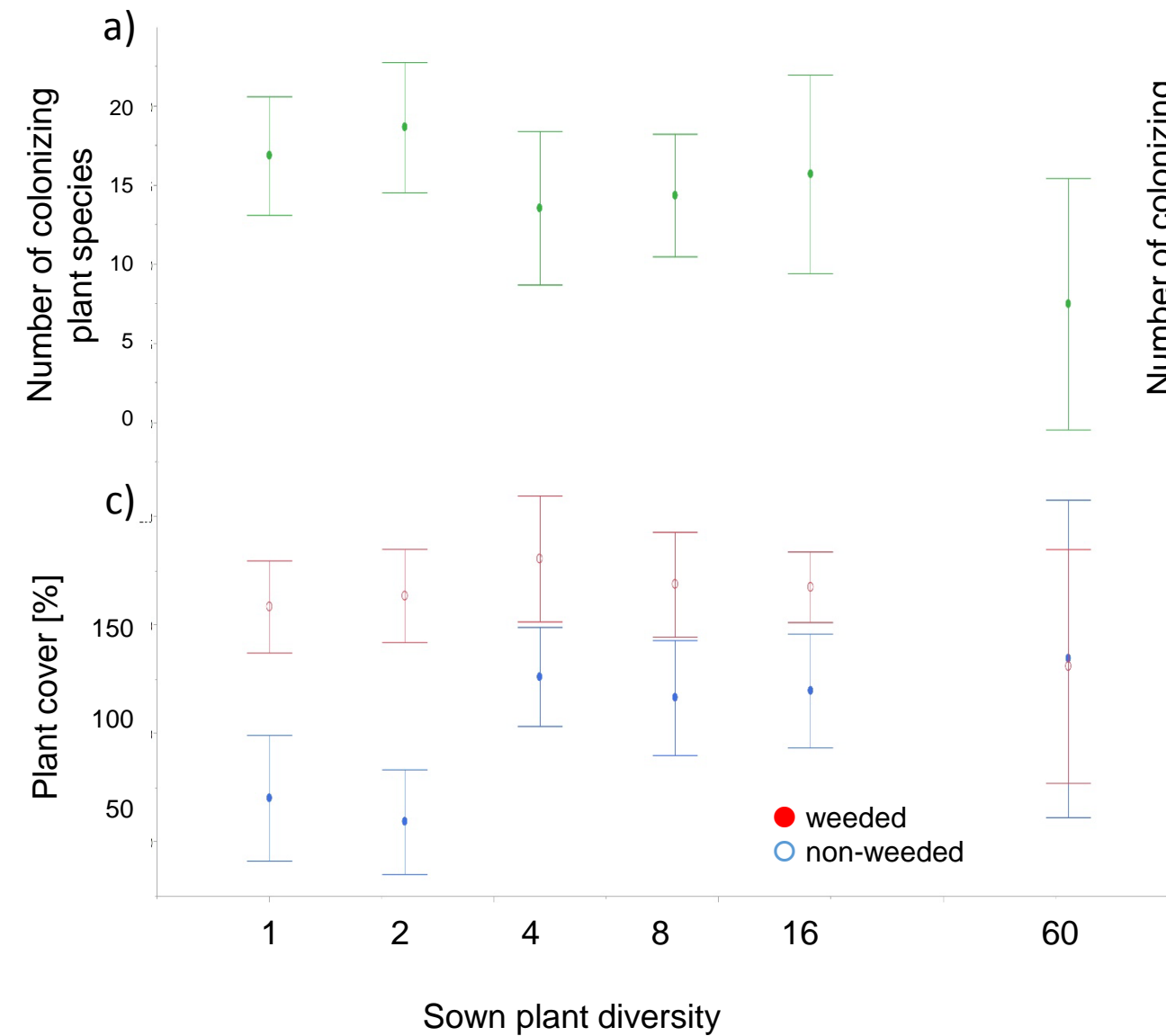

# Five years of plant colonization

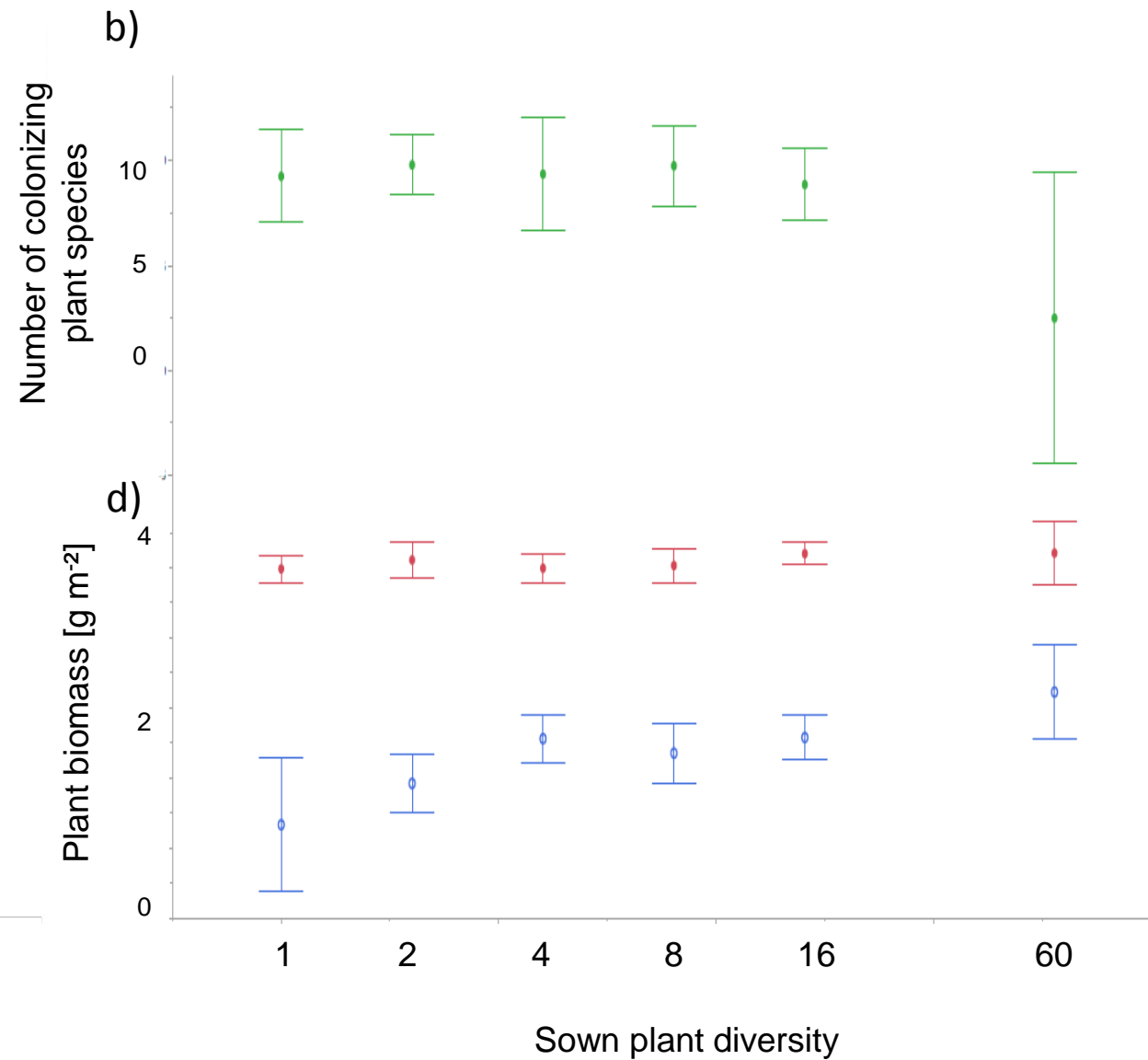

Supplement: Supplementary file 2 — 10.1186/s12898-016-0073-0 Plant colonization effects on plant cover and plant biomass. Mean values with confidence intervals of colonizing plant species after (a) two years and (b) five years. (c) Mean values with confidence intervals of plant cover [%] after two years and (d) plant biomass [g m−2] after five years. In c) and d) circles display plant cover and biomass of resident plant species of weeded subplots, respectively, and open circles display plant cover and biomass with resident plant species plus colonizing plant species of non-weeded subplots, respectively. [file 12898_2016_73_MOESM2_ESM.pdf]
