# Supplementary material for: Convergence of soil microbial properties after plant colonization of an experimental plant diversity gradient
Source: BMC Ecol. 2016 Apr 7;16:19. doi: 10.1186/s12898-016-0073-0 (PMC4825091; doi:10.1186/s12898-016-0073-0)
Supplement: Supplementary file 3 — 10.1186/s12898-016-0073-0 Plant colonization effects on soil microbial properties. Mean values with confidence intervals of basal respiration [µg O2 g−1 soil dry mass h−1] (a) two and (c) five years, and soil microbial biomass [µg C g−1 soil dry mass] (b) two and (d) five years after colonization by plant species. Circles display basal respiration and soil microbial biomass with resident plant species of weeded subplots, respectively, and open circles display basal respiration and soil microbial biomass with resident plant species plus colonizing plant species of non-weeded subplots, respectively. [file 12898_2016_73_MOESM3_ESM.pdf]

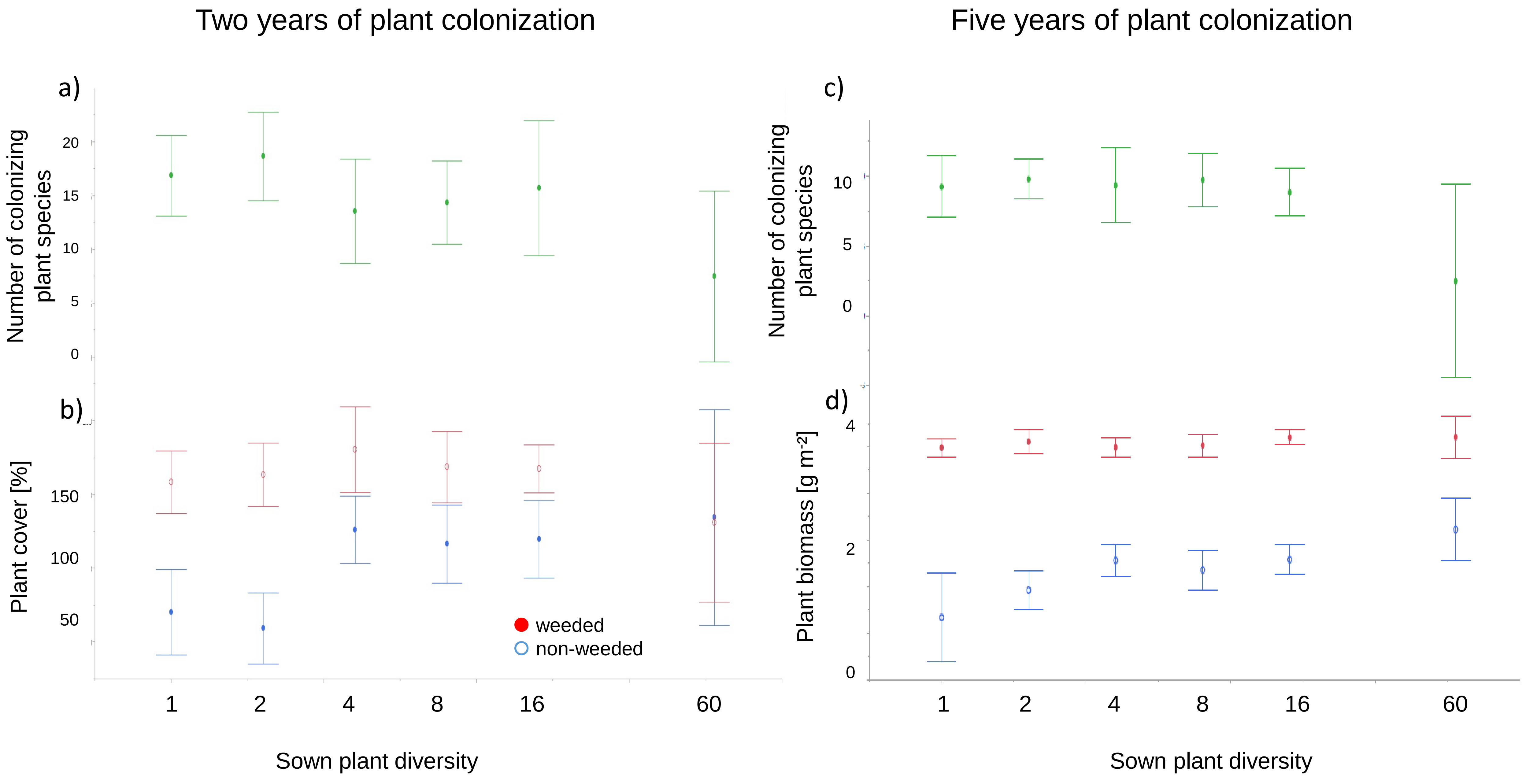

Two years of plant colonization
Five years of plant colonization
a)
b)
c)
d)
Number of colonizing plant species
Plant cover [%]
Plant biomass [g m-2]
0
5
10
15
20
50
100
150
2
4
weeded
non-weeded
1
2
4
8
16
60
Sown plant diversity

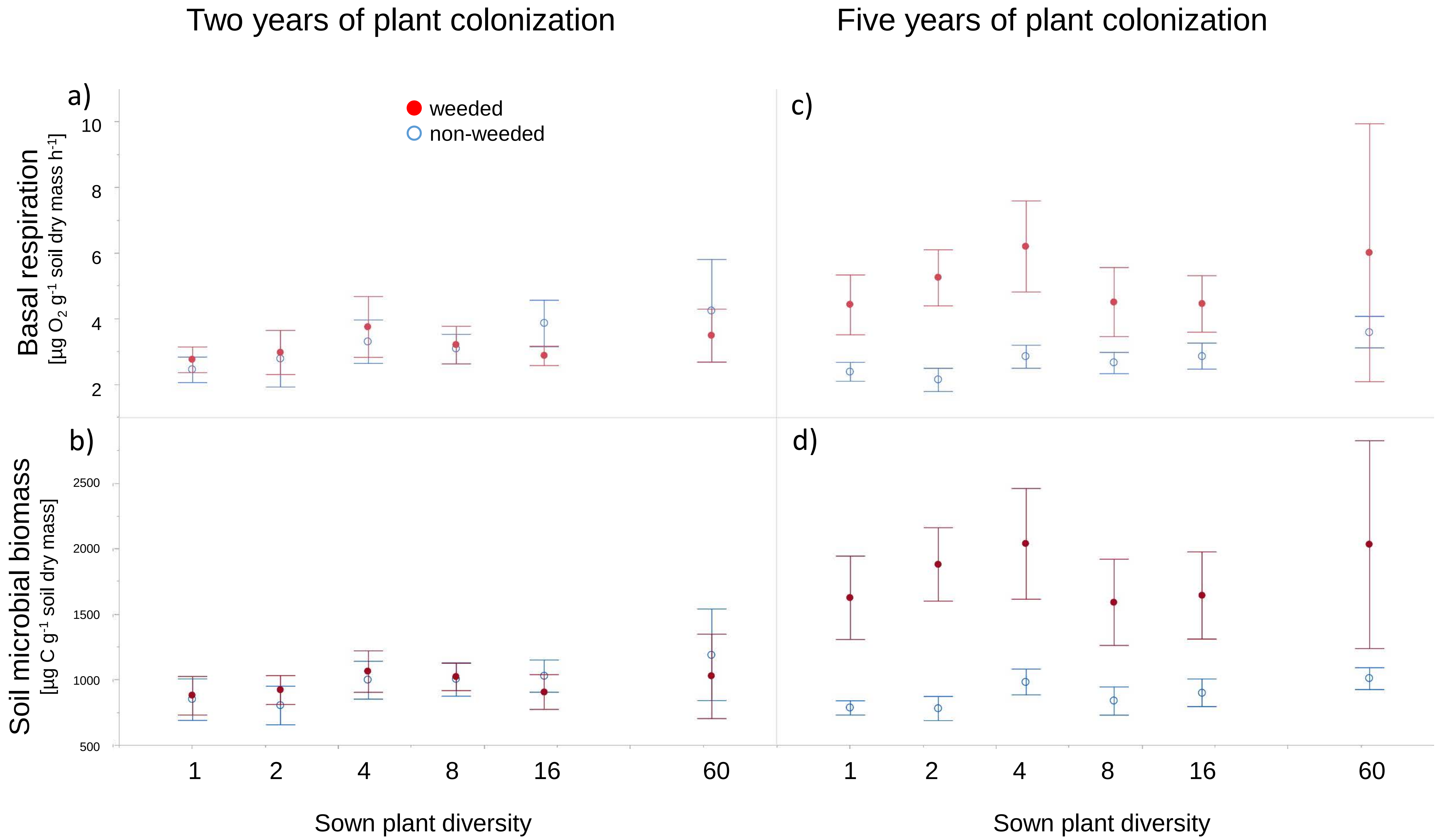

Two years of plant colonization
Five years of plant colonization
a)
b)
c)
d)
weeded
non-weeded
Basal respiration
[µg O2 g-1 soil dry mass h-1]
Soil microbial biomass
[µg C g-1 soil dry mass]
10
8
6
4
2
2500
2000
1500
1000
500
1
2
4
8
16
60
Sown plant diversity
Sown plant diversity
